# Womb Tipping

**Published:** 1886-01

**Authors:** 


"Womb Tipping."

Dr. Wm. Heath mentions the case of a woman who recently came to his office to be treated for sterility. She stated that she had been an actress, and, in order to prevent conception, had had her "womb tipped." She went to a man in New York who was an expert in this art, and who had operated on other women of her acquaintance. An instrument was passed into her uterus, causing some pain. This was followed by a slight discharge of blood. In a few days, she was able to go about her business as usual, and had never become pregnant, nor had any of her acquaintances on whom the operation had been performed. Dr. Heath found, on examination, that there was a marked retroflexion of the uterus.

